# A Preliminary Investigation of Brain Cannabinoid Receptor Type 1 (CB1R) Availability in Men with Opioid Use Disorder

**DOI:** 10.21203/rs.3.rs-7715611/v1

**Published:** 2025-10-14

**Authors:** ANAHITA BASSIR NIA, Ardavan Mohammad Aghaei, Jeremy Weleff, Julia Shi, Angelina Contreras, Mackenzie Griffin, Oluwole Jegede, Brian Pittman, Ilan Harpaz-Rotem, Ansel Hillmer, Deepak D’Souza

**Affiliations:** Yale University School of Medicine; Yale School of Medicine; Yale University; University of Michigan; Yale University

**Keywords:** Endocannabinoid, Cannabinoid Receptor type 1, Opioid Use Disorder, Opiates, Positron Emission Tomography, [11C]OMAR

## Abstract

The endocannabinoid (eCB) system has been proposed as a potential target for developing new medications for opioid use disorder (OUD). However, the status of the eCB system, specifically brain cannabinoid receptor type 1 (CB1R) in OUD, is unknown. In this study, CB1R availability was measured in males with OUD on stable opioid agonist treatment (OAT) (n = 10) versus healthy controls (HC) (n = 18), using High-Resolution Research Tomography (HRRT) and the CB1R-specific radiotracer, [^11^C]OMAR. The average volume of distribution (*V*_*T*_) across 13 regions was compared between the OUD and HC groups. Average *V*_*T*_ was 15% lower in OUD vs. HC subjects (p = 0.04). Lower *V*_*T*_ in OUD compared to HC was also observed in several corticolimbic areas. Within OUD no effects on CB1R availability were observed for treatment medication (methadone vs. buprenorphine), current stress levels, or antidepressant medication. No associations between the average *V*_*T*_ and duration of OAT treatment or time since the last illicit opioid use were observed. This preliminary study suggests lower CB1R availability in men with OUD. Larger studies are necessary to replicate these findings. Future research should also draw from a more heterogeneous population, particularly by incorporating females, to better assess the potential confounding and moderating clinical factors. If confirmed, the observed alterations in CB1R availability in OUD may provide a rationale for targeting the eCB system in the treatment of OUD.

## Introduction

An alarmingly high number of Americans continue to die from opioid overdose every day in the United States (1, 2). The National Survey on Drug Use and Health (NSDUH) reported that 9.9 million people misused opioids in 2018 in the US, and more than 117,000 individuals started using high-potency opioids for the first time (3). Despite widespread preventive and therapeutic efforts, the rates of drug overdose have increased fourfold over the past two decades, with opioids being involved in more than 68% of them (3). The current state-of-the-art treatment for opioid use disorder (OUD) primarily involves modulation of the opioid receptor system through Opioid Agonist Treatment (OAT) with medications such as methadone and buprenorphine, or opioid antagonist treatment with naltrexone (4, 5). However, OAT faces challenges with high rates of opioid relapse and treatment discontinuation among patients (6–10). More than 50% of individuals enrolled in these programs continue to use illicit opioids despite being in treatment, making effective OUD treatment challenging (11–13). There is a need to look for novel approaches that target systems beyond the existing FDA-approved opioid receptor-modulating medications for OUD. Increasing evidence suggests that the endocannabinoid (eCB) system could be a promising target (14–18).

The eCB system closely interacts with the endogenous opioid (opioidergic) system. Cannabinoid Receptor Type 1 (CB1R) and Mu Opioid Receptors (MOR) are both Gi/o-coupled receptors and are colocalized in several brain areas implicated in the pathophysiology of OUD (19–24). Furthermore, the eCB and opioidergic systems share similar functions in regulating stress and pain, and their agonistic activation yields similar results, including antinociception, sedation, hypotension, motor depression, and drug reward and reinforcement (25–27). Consequently, there is growing interest in exploring the role of the eCB system in OUD treatment (28, 29).

Preclinical studies have shown region-specific reductions in CB1R density, G-protein binding capacity, or mRNA levels in the cerebral cortex (30), hippocampus (31, 32), basolateral amygdala (32), and cerebellum (32), although not in all regions (32, 33). In addition, chronic morphine exposure in these preclinical models was associated with higher mRNA levels of Fatty-acid Amide Hydrolase (FAAH) and Monoacylglycerol Lipase (MAGL) (31), the two main eCB degrading enzymes, and lower levels of the principal endocannabinoid 2-Arachidonoylglycerol (2-AG) in several brain areas (34, 35). Consistent with preclinical findings, a recent PET imaging study using the FAAH-specific ligand showed that individuals with OUD have higher whole-brain FAAH levels (36). However, the status of CB1R in individuals with OUD has not yet been investigated.

The availability of reliable CB1R-specific radiotracers makes it possible to measure CB1R availability *in vivo* in humans. [^11^C]OMAR, a CB1R antagonist, has been successfully and reliably used in numerous studies to investigate CB1R availability in humans (37–42). The goal of this study was to measure CB1R *in vivo* in men with OUD on OAT, using [^11^C]OMAR tracer.

## Subjects and Methods

### Approvals:

This study was approved by the Yale University Institutional Review Board, the Yale Magnetic Resonance Research Center, and the Yale New Haven Hospital Radiation Drug Research Committee. All participants received detailed explanations, and written informed consent was obtained from each subject.

### Subjects:

Males with a diagnosis of OUD who were not using cannabis or other drugs were recruited. Participants were interviewed using the Structured Clinical Interview for DSM-5 (SCID-5), and individuals with concurrent major psychiatric disorders were excluded. Subjects were required to be under OAT with a stable dose of methadone or buprenorphine for at least one month, which was confirmed by contacting their OAT treatment centers or providers. Participants were excluded if they reported past-month use of any substances, including cannabis, and if they had a positive urine toxicology for any substance, except prescribed methadone or buprenorphine. Consistent with definitions from the Centers for Disease Control and Prevention and other studies, individuals who reported risky alcohol use (i.e., alcohol use exceeding 14 standard drinks per week) or heavy nicotine use (i.e., nicotine or tobacco use equivalent to one or more packs of cigarettes per day (PPD)) were excluded (43–45). Other exclusion criteria included claustrophobia, ferromagnetic metal in the body, a heart pacemaker, abnormal coagulation tests, and poor arterial access. To mitigate the potential effects of acute opioid intoxication and withdrawal, all PET scans were scheduled between 9 and 11 a.m., and participants were instructed to take their daily OAT dose only after completing the PET scan. Opioid craving and withdrawal symptoms were measured in the morning before the PET scan, and the PET scan was rescheduled if they had moderate or severe withdrawal symptoms. Male healthy controls (HC) with no psychiatric diagnosis based on SCID-5 and no substance use disorder (except nicotine) served as the comparison group.

### Measurements:

Nicotine dependence was assessed using the Fagerström Test for Nicotine Dependence (FTND), and perceived stress was evaluated using the Perceived Stress Scale (PSS). Opioid withdrawal symptoms were measured using the Clinical Opiate Withdrawal Scale (COWS) (cutoff score for moderate withdrawal symptoms > 7), and the intensity of opioid craving was measured using a Visual Analogue Scale (VAS), ranging from 0 to 100 (cutoff score for moderate craving > 30). The time since the last illicit opioid and cannabis use was recorded using the Drug History Questionnaire (DHQ), and the duration current OAT episode and current OAT dose was collected by the Methadone Questionnaire (MQ).

### Imaging:

#### MR Imaging:

Structural magnetic resonance imaging (3D MPRAGE) was conducted for participants using a Siemens 3-T Trio system (Siemens Medical Solutions, Malvern, Pennsylvania) with a circularly polarized head coil to exclude individuals with gross anatomical abnormalities and anatomically delineate regions of interest for PET analyses. The dimensions and voxel size of MR images were 256 × 256 × 176 and 0.98 × 0.98 × 1.0 mm3, respectively.

#### PET Imaging:

CB1R availability was measured using [^11^C]OMAR (46). PET data were acquired using a High-Resolution Research Tomograph (HRRT). Before PET scanning, two IV lines and an arterial catheter were placed. [^11^C]OMAR was prepared with high molar activity by previously described methods adapted to the TRACERlab FXC Pro automated synthesis module (GE Healthcare, Milwaukee, WI) (46). Image acquisition begun with a 6-minute transmission scan acquired for attenuation correction. Emission data acquisition was started with a bolus injection of up to 740 MBq [^11^C]OMAR and continued for at least 120 minutes. The head motion was continually tracked during the acquisition of PET data with a Vicra marker-based tracking system (NDI Systems, Waterloo, Ontario). Since no reference region existed for [^11^C]OMAR, arterial blood samples were collected contralaterally from the radiotracer administration arm during the PET scan to measure the plasma input function and High-Performance Liquid Chromatography (HPLC) analysis of radioactive metabolites. Radioactivity in plasma was measured with a gamma counter. The column-switching HPLC analysis method was used to assess radioactive metabolites and unchanged parent compounds (47). The measured input function was the product of the radioactivity concentration in plasma and the unchanged parent fraction.

#### Image Processing and Analysis:

To ensure blind data processing and analysis, a pseudo-randomized subject identifier devoid of diagnostic and demographic information was created and assigned to each participant upon uploading data into our database. Image analysts only saw this subject identifier during pre-processing and analyses. List mode data was reconstructed using MOLAR (48), with attenuation, normalization, and motion corrections. Early PET image data were registered to the subject’s T1-weighted MR image. The T1-weighted MR image was non-linearly registered to MNI space for the region of interest (ROI) identification using the Anatomical Automatic Labeling (AAL) atlas (49). Predetermined ROIs included the following thirteen regions: amygdala, caudate, cerebellum, anterior cingulate (AC), posterior cingulate (PC), frontal, hippocampus, insula, occipital cortex, parietal cortex, putamen, temporal, and thalamus. The nucleus accumbens and ventral tegmental area were not included due to challenges in imaging with the [^11^C]OMAR radiotracer given their small size, anatomical location, and the tracer’s characteristics. Since OUD is associated with significantly lower gray matter volumes (50–52), this was accounted for with partial volume correction (53), as previously implemented (54, 55). [^11^C]OMAR *V*_T_ was estimated using the metabolite-corrected arterial plasma input function with multilinear 1 analysis (MA1; t*=30 min) (56), which has good test-retest reproducibility with mean absolute deviations of 7.3–10.0%.

### Statistical Analysis:

All data were summarized descriptively, and normality was assessed using normal probability plots and Kolmogorov–Smirnov test statistics. Consistent with prior PET imaging studies employing the [^11C]OMAR radiotracer in psychiatric populations (41, 42, 57), the primary outcome was defined as the average CB1R availability, calculated as the mean value across regions of interest. The primary effect of interest was the main effect of group, testing for overall differences in CB1R availability between groups.

For the primary analysis, composite VT levels were compared between healthy controls (HC) and individuals with opioid use disorder (OUD) using independent, two-sided t-tests. Given the small sample size, additional exploratory analyses were performed to examine region-specific differences in binding between groups using independent t-tests. For these analyses, both unadjusted and false discovery rate (FDR)–corrected p-values are reported.

Further exploratory analyses employed linear mixed models (LMMs) to evaluate the main and interaction effects of potential covariates within the OUD group, including OAT medication, antidepressant use, FTND, and PSS scores. All analyses were conducted using SAS software, version 9.4 (SAS Institute Inc., Cary, NC).

## Results

A total of 10 male individuals with OUD and 18 male HC participants were studied (Table 1). No significant differences were found in sociodemographic characteristics. On the scan day, craving (4.0 ± 9.7 [SD]) and withdrawal (0.8 ± 1.5) scores were low among participants with OUD. As per the inclusion criteria, participants did not have concurrent active major psychiatric or substance use disorders. One subject had current symptoms of depression due to chronic dysthymia. Subjects with OUD reported more nicotine use (Table 1) and treatment with antidepressant medications for a history of depression and other psychiatric disorders (supplemental Table 1).

### CB1R availability in individuals with OUD compared to HC.

**Figure 1:**
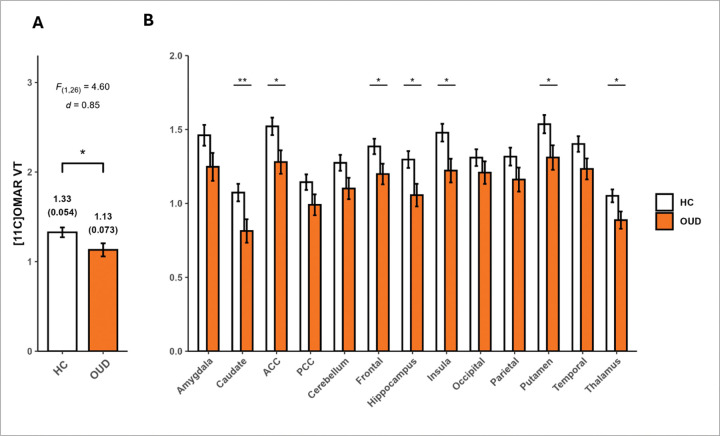
LS means of [^11^C]OMAR V_T_ averaged across brain regions (a) and [^11^C]OMAR V_T_ LS mean in each region b) in men with opioid use disorder (OUD) and Healthy Controls (HC). LS means presented were estimated from the same LMM with group, region, and their interaction as predictors.

Lower global (composite across regions) CB1R availability was observed in participants with OUD compared to HC (group effect: *F*_*(1,26)*_ = 4.6, *p*= 0.04, difference = −14.7%, *d*= 0.85) (Figure 1a). The exploratory analyses showed a significant group-by-region interaction (*F*_(12,312)_= 2.42, *p*= 0.005), where group differences were observed in some but not all regions. Specifically, group effects were observed in several corticolimbic areas, including AC (−15.87%, *F*_(1,312)_= 5.91, *p*= 0.016, *d*=0.96), caudate (−24.21%, *F*_(1,312)_= 6.85, *p*= 0.009, *d*=1.03), frontal (−13.49%, *F*_(1,312)_= 4.60, *p*= 0.033, *d*=0.84), hippocampus (−18.57%, *F*_(1,312)_= 6.34, *p*= 0.012, *d*=0.99), insula (−17.34%, *F*_(1,312)_= 6.56, *p*= 0.011, *d*=1.01), putamen (−14.71%, *F*_(1,312)_= 4.76, *p*= 0.030, *d*=0.86), and thalamus (−15.63%, *F*_(1,312)_= 5.06, *p*= 0.025, *d*=0.89) (Table 2 and Figure 1b). In addition to these group differences, there was a highly significant group by region interaction, which was substantially driven by a greater overall region effect within HC (F_(12,312)_= 71.7, p<.0001), compared to OUD (F_(12, 312)_= 39.5, p<.0001). That is, although both effects are highly significant, the F-statistic for HC is >80% higher than that for OUD. Pair-wise comparisons between regions within each group are not of primary interest and thus not reported. Supplementary Table S2 summarizes the result of a similar LMM model with age added as a covariate.

### Exploratory analysis of the impact of potential covariates on CB1R availability in OUD.

#### • Methadone vs. buprenorphine treatment:

There were no significant differences (*F*_(1,8)_ = 0.52, p = 0.49) in overall CB1R availability between those receiving methadone (n = 7; mean dose: 80.7, SD: 32.2 mg) and those receiving buprenorphine (n = 3; mean dose: 22.7, SD: 3.2 mg) (Table S3). No associations were observed between OAT dose and CB1R availability.

#### • Current stress level:

No significant associations existed between current stress levels in the OUD group, as measured using PSS, and CB1R availability (Spearman’s r= 0.07, p=0.84).

#### • Nicotine use:

Among individuals with any nicotine use (n=6), no associations were observed between FTND scores and CB1R availability (Spearman’s r=0.09, p=0.87).

#### • Antidepressant medications:

Among individuals with OUD, there were no significant differences in overall CB1R availability between those taking (1.11 ± 0.042) and those not taking (1.19 ± 0.064) antidepressant medication (*F*_(1,8)_ = 1.23, p = 0.30) (Table S4).

## Discussion

To our knowledge, this is the first study to measure CB1R availability in individuals with OUD *in vivo*. Males with OUD (on stable OAT) showed 15% lower global CB1R availability compared to healthy male individuals. This finding aligns with several (30–32), but not all (32, 33), animal studies showing lower CB1R density or mRNA levels in several brain regions.

The findings of lower CB1R availability are intriguing in light of higher FAAH levels reported in individuals with OUD (36). Higher FAAH activity is associated with lower AEA levels (57). The lower CB1R availability observed in our study, combined with high FAAH activity in individuals with OUD reported elsewhere (36), suggests a lower eCB tone in males with OUD.

There is substantial evidence of extensive overlap between the eCB and opioid systems that may explain why exposure to opioids could lead to lower CB1R levels. When administered acutely, cannabinoids and CB1R agonists increase endogenous opioid release (endorphin and enkephalin) (58, 59), and MOR expression (60, 61). Similarly, acute administration of opioids alters eCB levels (62, 63), and CB1R expression (61, 64, 65), and functionality (61). Moreover, the colocalization of CB1R and MOR in several brain areas is the basis of functional interactions (19, 20). Increasing evidence suggests that there is bidirectional modulation of the rewarding and reinforcing properties of opioids and cannabinoids (22). Preclinical studies of chronic opioid exposure have shown region-specific reductions in CB1R density, G-protein binding capacity, or mRNA levels in the cerebral cortex (30), hippocampus (31, 32), basolateral amygdala (32), and cerebellum (32), in addition to higher levels of FAAH and MAGL mRNA (31), and lower levels of 2-AG in several brain regions (34, 35).

The low CB1R availability in individuals with OUD suggests that the eCB system could potentially serve as a new target in OUD treatment (27, 66–68). Preclinical studies demonstrated that eCB modulation reduces the reinforcing effects of opioids (15, 69), opioid-seeking behaviors (70, 71), and opioid withdrawal symptoms (72). Similarly, human studies reported that targeting the eCB system is effective in attenuating opioid craving (73). Moreover, positive effects of cannabinoids or eCB modulators in the treatment of OUD comorbidities such as chronic pain have been reported (74–81). Some but not all observational studies suggest that cannabinoids may have opioid-sparing effects (82), while experimental studies suggest mixed results (83). Although research on the effects of cannabis or its principal active constituent, delta-9-tetrahydrocannabinol (THC), on OUD treatment remains inconclusive (84), new investigational eCB modulators, such as FAAH and MAGlipase inhibitors, are now available to target the eCB system (85). Current evidence shows promising therapeutic effects of FAAH inhibitors in nicotine and cannabis dependence (86, 87). A significant advantage of these compounds is their ability to modulate the eCB function regionally, causing minimal, if any, CB1R downregulation that is typically induced by direct CB1R agonists such as THC (41). Moreover, FAAH inhibitors have low abuse liability, which is a significant benefit compared to cannabis or THC (88). Further studies are required to investigate the potential therapeutic effects of eCB modulators in treating OUD.

The involvement of the eCB system in stress regulation and reward processing has been extensively reported (18, 89–94). Moreover, low CB1R availability was reported in other substance use disorders, including cannabis use disorder (41), alcohol use disorder (95), and tobacco use disorder (96). It remains unclear whether this reduced CB1R availability is drug-specific or reflects broader deficits in reward processing and heightened stress reactivity in addiction, regardless of substance type. Further investigations are also required to determine if lower CB1R in individuals with OUD precedes the development of OUD, or is a consequence of long-term opioid use, and to investigate other factors potentially involved in these alterations. Future research should consider comparing the CB1R availability in individuals with OUD who are not on OAT to assess if similar low CB1R availability exists in individuals with OUD regardless of OAT. Moreover, measuring CB1R availability before and after opioid abstinence periods would illuminate the impact of active opioid use on CB1R. Furthermore, assessing CB1R availability in individuals prescribed opioids without OUD could differentiate the general effects of opioid use on CB1R from those specifically associated with OUD.

This study has certain limitations that should be considered when interpreting the results. Only male participants with OUD were recruited in this study. Existing literature indicates sex differences in the eCB system both at baseline and in the context of other psychiatric disorders (97–106). Thus, future studies should explore the eCB status in females to better understand these potential sex differences. The limited sample size reduces overall statistical power, and specifically limits estimating potential covariate effects– future studies with larger samples and a comparable number of participants receiving methadone or buprenorphine treatment are necessary to replicate these preliminary findings. While there were no significant group differences in age, the OUD group was older, with a few participants over 65. Additionally, our sample was unbalanced regarding nicotine dependence. A larger study with a balanced age and nicotine dependence distribution is necessary to parse out the effect of opioid exposure from potential confounders. Furthermore, only OUD participants who were stable on OAT were included to minimize the effects of opioid withdrawal and intoxication on CB1R availability. Further research is necessary to determine if individuals with OUD who are not receiving OAT exhibit similar low CB1R availability. Lastly, our study excluded individuals using other substances, including cannabis. This decision was critical for the interpretability of the data, given strong evidence that cannabis use is associated with lower availability of CB1R (41). However, excluding other substance use may limit the generalizability of our results to the broader population of OUD individuals with co-occurring use of other substances. Future studies should consider including individuals with OUD and concurrent substance use disorders to enhance the generalizability of the findings.

In summary, our results provide preliminary evidence of lower global CB1R availability (composite across regions) in males with OUD in OAT treatment. Future studies are required to determine the clinical implications of low CB1R in males with OUD, and the possibility of targeting the eCB system in treating OUD.

## Supplementary Material

Supplementary Files

This is a list of supplementary files associated with this preprint. Click to download.

• TheCB1RavailabilityinOUDSupplements.docx

## Figures and Tables

**Table 1:** Demographic characteristics of participants

	OUD (N=10)	HC (N=18)	*p* vlaue^1^
**Age; mean(SD)**	56.82 (10.93)	49.22 (8.46)	0.08
**BMI; mean(SD)**	31.58 (5.77)	29.77 (5.77)	0.43
**Race**			0.11
**African American; n(%)**	1	3	
**Caucasian; n(%)**	9	10	
**Other/Unknown; n(%)**	0	5	
**Ethnicity**			0.06
**Non-Hispanic or Latino**	10	12	
**Hispanic or Latino**	0	0	
**Other/Unknown**	0	6	
**Tracer dose; MBq; mean(SD)**	429.20 (180.93)	556.11 (120.62)	0.07
**Nicotine dependence^2^; n (%)**	6 (60%)	1 (5.6%)	<0.01
**FTND Score^3^; mean (SD)**	2.3 (1.2)	4.0 (-)	-
**PSS score; mean (SD)**	10.7 (7.2)	-	-
**COWS score^4^; mean (SD)**	0.8 (1.5)	-	-
**Craving VAS score^4^; mean (SD)**	4.0 (9.7)	-	-
**Methadone; n (%)**	7 (70%)	-	-
**Methadone dose; means (SD)**	80.7 (32.2)	-	-
**Buprenorphine; n (%)**	3 (30%)	-	-
**Buprenorphine; dose (SD)**	22.7 (2.3)	-	-
**Last illicit opioid use, months; mean (SD)**	88.7 (90.07)		
**Last cannabis use, months; mean (SD)**	275.37 (180.31)		
**Past 12 months alcohol use disorders, in remission; n (%)**	1 (10%)		
**Current antidepressant use; n (%)**	6 (60%)	-	-
**Current benzodiazepine use; n (%)**	0 (0%)	-	-

Abbreviations: BMI, Body Mass Index; COWS, Clinical Opiate Withdrawal Scale; FTND, Fagerström Test for Nicotine Dependence; HC, Healthy Control; OUD, Opioid Use Disorder; PSS; Perceived Stress Scale; VAS, Visual Analog Scale.

1 Based on the T-test for continuous and Chi-square or Fisher exact test for categorical variables.

2 Defined as FTND score of greater than zero.

3 In participants with nicotine dependence.

4 Before the tracer injection.

**Table 2:** Estimated regional LS means and SEs of CB1R availability with post hoc comparisons based on the LMM

	LS mean (SE)		Contrast %	*F_(1,312)_*	*d*	*P_Uncorrected_*
	OUD	HC				
**Anterior Cingulate**	1.28 (0.08)	1.52 (0.06)	−15.87%	5.91	0.96	**0.016**
**Amygdala**	1.25 (0.09)	1.46 (0.07)	−14.59%	3.27	0.71	0.071
**Caudate**	0.81 (0.08)	1.07 (0.06)	−24.21%	6.85	1.03	**0.009**
**Cerebellum**	1.10 (0.07)	1.27 (0.05)	−13.61%	3.70	0.76	0.055
**Frontal**	1.20 (0.07)	1.38 (0.05)	−13.49%	4.6	0.84	**0.033**
**Hippocampus**	1.06 (0.08)	1.30 (0.06)	−18.57%	6.34	0.99	**0.012**
**Insula**	1.22 (0.08)	1.48 (0.06)	−17.34%	6.56	1.01	**0.011**
**Occipital**	1.21 (0.08)	1.31 (0.06)	−7.74%	1.14	0.42	0.286
**Parietal**	1.16 (0.08)	1.32 (0.06)	−11.80%	2.37	0.61	0.125
**Posterior Cingulate**	0.99 (0.07)	1.14 (0.05)	−13.36%	3.00	0.68	0.084
**Putamen**	1.31 (0.08)	1.54 (0.06)	−14.71%	4.76	0.86	**0.030**
**Temporal**	1.23 (0.07)	1.40 (0.05)	−12.04%	3.64	0.75	0.057
**Thalamus**	0.89 (0.06)	1.05 (0.04)	−15.63%	5.06	0.89	**0.025**

## References

[1] Wide-ranging online data for epidemiologic research (WONDER). Atlanta GC, National Center for Health Statistics; 2017. Available at http://wonder.cdc.gov.

[2] National Center for Health Statistics (2025): U.S. Overdose Deaths Decrease in 2023, First Time Since 2018. National Center for Health Statistics,.

[3] U.S. Department of Health and Human Services, Substance Abuse and Mental Health Services Administration, Center for Behavioral Health Statistics and Quality (2020): National Survey on Drug Use and Health 2019 (NSDUH-2019-DS0001).

[4] Kampman K, Jarvis M (2015): American Society of Addiction Medicine (ASAM) National Practice Guideline for the Use of Medications in the Treatment of Addiction Involving Opioid Use. J Addict Med. 9:358–367. 26406300 10.1097/ADM.0000000000000166 PMC4605275

[5] The ASAM National Practice Guideline for the Treatment of Opioid Use Disorder: 2020 Focused Update. J Addict Med. 14:1–91. 10.1097/ADM.0000000000000633 32511106

[6] Nosyk B, Marsh DC, Sun H, Schechter MT, Anis AH (2010): Trends in methadone maintenance treatment participation, retention, and compliance to dosing guidelines in British Columbia, Canada: 1996–2006. J Subst Abuse Treat. 39:22–31. 20418051 10.1016/j.jsat.2010.03.008

[7] Lo A, Kerr T, Hayashi K, Milloy MJ, Nosova E, Liu Y, (2018): Factors associated with methadone maintenance therapy discontinuation among people who inject drugs. J Subst Abuse Treat. 94:41–46. 30243416 10.1016/j.jsat.2018.08.009 PMC6375706

[8] Hser YI, Saxon AJ, Huang D, Hasson A, Thomas C, Hillhouse M, (2014): Treatment retention among patients randomized to buprenorphine/naloxone compared to methadone in a multi-site trial. Addiction. 109:79–87. 23961726 10.1111/add.12333 PMC3947022

[9] Smyth BP, Barry J, Keenan E, Ducray K (2010): Lapse and relapse following inpatient treatment of opiate dependence. Ir Med J. 103:176–179. 20669601

[10] Darker CD, Ho J, Kelly G, Whiston L, Barry J (2016): Demographic and clinical factors predicting retention in methadone maintenance: results from an Irish cohort. Ir J Med Sci. 185:433–441. 26026953 10.1007/s11845-015-1314-5

[11] Strain EC, Bigelow GE, Liebson IA, Stitzer ML (1999): Moderate- vs high-dose methadone in the treatment of opioid dependence: a randomized trial. JAMA. 281:1000–1005. 10086434 10.1001/jama.281.11.1000

[12] Calabria B, Degenhardt L, Briegleb C, Vos T, Hall W, Lynskey M, (2010): Systematic review of prospective studies investigating “remission” from amphetamine, cannabis, cocaine or opioid dependence. Addict Behav. 35:741–749. 20444552 10.1016/j.addbeh.2010.03.019

[13] Vaillant GE (1973): A 20-year follow-up of New York narcotic addicts. Arch Gen Psychiatry. 29:237–241. 4741515 10.1001/archpsyc.1973.04200020065009

[14] Martínez-Rivera A, Fetcho RN, Birmingham L, Xu J, Yang R, Foord C, (2024): Elevating levels of the endocannabinoid 2-arachidonoylglycerol blunts opioid reward but not analgesia. Sci Adv. 10:eadq4779. 39612328 10.1126/sciadv.adq4779 PMC11606496

[15] Wilkerson JL, Ghosh S, Mustafa M, Abdullah RA, Niphakis MJ, Cabrera R, (2017): The endocannabinoid hydrolysis inhibitor SA-57: Intrinsic antinociceptive effects, augmented morphine-induced antinociception, and attenuated heroin seeking behavior in mice. Neuropharmacology. 114:156–167. 27890602 10.1016/j.neuropharm.2016.11.015 PMC5289715

[16] Oliva I, Kazi F, Cantwell LN, Thakur GA, Crystal JD, Hohmann AG (2024): Negative allosteric modulation of CB1 cannabinoid receptor signalling decreases intravenous morphine self-administration and relapse in mice. Addict Biol. 29:e13429. 39109814 10.1111/adb.13429 PMC11304470

[17] Iyer V, Saberi SA, Pacheco R, Sizemore EF, Stockman S, Kulkarni A, (2024): Negative allosteric modulation of CB(1) cannabinoid receptor signaling suppresses opioid-mediated tolerance and withdrawal without blocking opioid antinociception. Neuropharmacology. 257:110052. 38936657 10.1016/j.neuropharm.2024.110052 PMC11261750

[18] Gamble MC, Miracle S, Williams BR, Logan RW (2024): Endocannabinoid agonist 2-arachidonoylglycerol differentially alters diurnal activity and sleep during fentanyl withdrawal in male and female mice. Pharmacol Biochem Behav. 240:173791. 38761993 10.1016/j.pbb.2024.173791 PMC11166043

[19] Scavone JL, Mackie K, Van Bockstaele EJ (2010): Characterization of cannabinoid-1 receptors in the locus coeruleus: relationship with mu-opioid receptors. Brain Res. 1312:18–31. 19931229 10.1016/j.brainres.2009.11.023 PMC2835571

[20] Rodriguez JJ, Mackie K, Pickel VM (2001): Ultrastructural localization of the CB1 cannabinoid receptor in mu-opioid receptor patches of the rat Caudate putamen nucleus. J Neurosci. 21:823–833. 11157068 10.1523/JNEUROSCI.21-03-00823.2001 PMC6762333

[21] Lopez-Moreno JA, Lopez-Jimenez A, Gorriti MA, de Fonseca FR (2010): Functional interactions between endogenous cannabinoid and opioid systems: focus on alcohol, genetics and drug-addicted behaviors. Curr Drug Targets. 11:406–428. 20196742 10.2174/138945010790980312

[22] Ahmad T, Lauzon NM, de Jaeger X, Laviolette SR (2013): Cannabinoid transmission in the prelimbic cortex bidirectionally controls opiate reward and aversion signaling through dissociable kappa versus mu-opiate receptor dependent mechanisms. J Neurosci. 33:15642–15651. 24068830 10.1523/JNEUROSCI.1686-13.2013 PMC6618460

[23] Wills KL, DeVuono MV, Limebeer CL, Vemuri K, Makriyannis A, Parker LA (2017): CB(1) receptor antagonism in the bed nucleus of the stria terminalis interferes with affective opioid withdrawal in rats. Behav Neurosci. 131:304–311. 28714716 10.1037/bne0000201

[24] Parolaro D, Rubino T, Vigano D, Massi P, Guidali C, Realini N (2010): Cellular mechanisms underlying the interaction between cannabinoid and opioid system. Curr Drug Targets. 11:393–405. 20017730 10.2174/138945010790980367

[25] Massi P, Vaccani A, Romorini S, Parolaro D (2001): Comparative characterization in the rat of the interaction between cannabinoids and opiates for their immunosuppressive and analgesic effects. J Neuroimmunol. 117:116–124. 11431011 10.1016/s0165-5728(01)00323-x

[26] Maldonado R, Valverde O (2003): Participation of the opioid system in cannabinoid-induced antinociception and emotional-like responses. Eur Neuropsychopharmacol. 13:401–410. 14636956 10.1016/j.euroneuro.2003.08.001

[27] Scavone JL, Sterling RC, Van Bockstaele EJ (2013): Cannabinoid and opioid interactions: implications for opiate dependence and withdrawal. Neuroscience. 248:637–654. 23624062 10.1016/j.neuroscience.2013.04.034 PMC3742578

[28] Suzuki J, Weiss RD (2021): Cannabinoids for the Treatment of Opioid Use Disorder: Where is the Evidence? J Addict Med. 15:91–92. 32909980 10.1097/ADM.0000000000000711 PMC7986228

[29] Blessing E, Virani S, Rotrosen J (2020): Clinical Trials for Opioid Use Disorder. Handb Exp Pharmacol. 258:167–202. 31889218 10.1007/164_2019_304

[30] Gonzalez S, Schmid PC, Fernandez-Ruiz J, Krebsbach R, Schmid HH, Ramos JA (2003): Region-dependent changes in endocannabinoid transmission in the brain of morphine-dependent rats. Addict Biol. 8:159–166. 12850774 10.1080/1355621031000117383

[31] Li W, Zhang CL, Qiu ZG (2017): Differential expression of endocannabinoid system-related genes in the dorsal hippocampus following expression and reinstatement of morphine conditioned place preference in mice. Neurosci Lett. 643:38–44. 28192193 10.1016/j.neulet.2017.02.025

[32] Gonzalez S, Fernandez-Ruiz J, Sparpaglione V, Parolaro D, Ramos JA (2002): Chronic exposure to morphine, cocaine or ethanol in rats produced different effects in brain cannabinoid CB(1) receptor binding and mRNA levels. Drug Alcohol Depend. 66:77–84. 11850139 10.1016/s0376-8716(01)00186-7

[33] Rubino T, Tizzoni L, Viganò D, Massi P, Parolaro D (1997): Modulation of rat brain cannabinoid receptors after chronic morphine treatment. Neuroreport. 8:3219–3223. 9351646 10.1097/00001756-199710200-00007

[34] Vigano D, Grazia Cascio M, Rubino T, Fezza F, Vaccani A, Di Marzo V, (2003): Chronic morphine modulates the contents of the endocannabinoid, 2-arachidonoyl glycerol, in rat brain. Neuropsychopharmacology. 28:1160–1167. 12637958 10.1038/sj.npp.1300117

[35] Vigano D, Valenti M, Cascio MG, Di Marzo V, Parolaro D, Rubino T (2004): Changes in endocannabinoid levels in a rat model of behavioural sensitization to morphine. Eur J Neurosci. 20:1849–1857. 15380006 10.1111/j.1460-9568.2004.03645.x

[36] Shyu C, Butler K, Tyndale RF, Smith A, Tardelli VS, Kloiber S, (2024): Investigating Endocannabinoid Metabolism in Opioid Treated Chronic Pain Patients With and Without Opioid Use Disorder: A PET Study With Fatty Acid Amide Hydrolase Radioligand [C-11]CURB. Biological Psychiatry. 95:S113.

[37] Wong DF, Kuwabara H, Horti AG, Raymont V, Brasic J, Guevara M, (2010): Quantification of cerebral cannabinoid receptors subtype 1 (CB1) in healthy subjects and schizophrenia by the novel PET radioligand [11C]OMAR. Neuroimage. 52:1505–1513. 20406692 10.1016/j.neuroimage.2010.04.034 PMC6580862

[38] Normandin MD, Zheng MQ, Lin KS, Mason NS, Lin SF, Ropchan J, (2015): Imaging the cannabinoid CB1 receptor in humans with [11C]OMAR: assessment of kinetic analysis methods, test-retest reproducibility, and gender differences. J Cereb Blood Flow Metab. 35:1313–1322. 25833345 10.1038/jcbfm.2015.46 PMC4528005

[39] Tsujikawa T, Zoghbi SS, Hong J, Donohue SR, Jenko KJ, Gladding RL, (2014): In vitro and in vivo evaluation of (11)C-SD5024, a novel PET radioligand for human brain imaging of cannabinoid CB1 receptors. Neuroimage. 84:733–741. 24076222 10.1016/j.neuroimage.2013.09.043 PMC3849221

[40] Neumeister A, Normandin MD, Pietrzak RH, Piomelli D, Zheng MQ, Gujarro-Anton A, (2013): Elevated brain cannabinoid CB1 receptor availability in post-traumatic stress disorder: a positron emission tomography study. Mol Psychiatry. 18:1034–1040. 23670490 10.1038/mp.2013.61 PMC3752332

[41] D’Souza DC, Cortes-Briones JA, Ranganathan M, Thurnauer H, Creatura G, Surti T, (2016): Rapid Changes in CB1 Receptor Availability in Cannabis Dependent Males after Abstinence from Cannabis. Biol Psychiatry Cogn Neurosci Neuroimaging. 1:60–67. 10.1016/j.bpsc.2015.09.008 PMC4742341 29560896

[42] Ranganathan M, Cortes-Briones J, Radhakrishnan R, Thurnauer H, Planeta B, Skosnik P, (2016): Reduced Brain Cannabinoid Receptor Availability in Schizophrenia. Biol Psychiatry. 79:997–1005. 26432420 10.1016/j.biopsych.2015.08.021 PMC4884543

[43] Boersma P, Villarroel MA, Vahratian A (2020): Heavy Drinking Among U.S. Adults, 2018. Centers for Disease Control and Prevention: Centers for Disease Control and Prevention.

[44] Tarasi B, Cornuz J, Clair C, Baud D (2022): Cigarette smoking during pregnancy and adverse perinatal outcomes: a cross-sectional study over 10 years. BMC Public Health. 22:2403. 36544092 10.1186/s12889-022-14881-4 PMC9773571

[45] Pierce JP, Messer K, White MM, Cowling DW, Thomas DP (2011): Prevalence of heavy smoking in California and the United States, 1965–2007. Jama. 305:1106–1112. 21406647 10.1001/jama.2011.334

[46] Horti AG, Fan H, Kuwabara H, Hilton J, Ravert HT, Holt DP, (2006): 11C-JHU75528: a radiotracer for PET imaging of CB1 cannabinoid receptors. J Nucl Med. 47:1689–1696. 17015906

[47] Hilton J, Yokoi F, Dannals RF, Ravert HT, Szabo Z, Wong DF (2000): Column-switching HPLC for the analysis of plasma in PET imaging studies. Nucl Med Biol. 27:627–630. 11056380 10.1016/s0969-8051(00)00125-6

[48] Carson RE, Barker WC, Liow J-S, Johnson CA (2003): Design of a motion-compensation OSEM list-mode algorithm for resolution-recovery reconstruction for the HRRT. Nuclear Science Symposium Conference Record, 2003 IEEE: IEEE, pp 3281–3285.

[49] Tzourio-Mazoyer N, Landeau B, Papathanassiou D, Crivello F, Etard O, Delcroix N, (2002): Automated anatomical labeling of activations in SPM using a macroscopic anatomical parcellation of the MNI MRI single-subject brain. Neuroimage. 15:273–289. 11771995 10.1006/nimg.2001.0978

[50] Lin HC, Wang PW, Wu HC, Ko CH, Yang YH, Yen CF (2018): Altered gray matter volume and disrupted functional connectivity of dorsolateral prefrontal cortex in men with heroin dependence. Psychiatry Clin Neurosci. 72:435–444. 29582514 10.1111/pcn.12655

[51] Schmidt A, Vogel M, Baumgartner S, Wiesbeck GA, Lang U, Borgwardt S, (2021): Brain volume changes after long-term injectable opioid treatment: A longitudinal voxel-based morphometry study. Addict Biol. 26:e12970. 33000891 10.1111/adb.12970

[52] Shi H, Liang Z, Chen J, Li W, Zhu J, Li Y, (2020): Gray matter alteration in heroin-dependent men: An atlas-based magnetic resonance imaging study. Psychiatry Res Neuroimaging. 304:111150. 32717665 10.1016/j.pscychresns.2020.111150 PMC8170872

[53] Giovacchini G, Lerner A, Toczek MT, Fraser C, Ma K, DeMar JC, (2004): Brain Incorporation of 11C-Arachidonic Acid, Blood Volume, and Blood Flow in Healthy Aging: A Study With Partial-Volume Correction. Journal of Nuclear Medicine. 45:1471–1479. 15347713

[54] Hillmer AT, Sandiego CM, Hannestad J, Angarita GA, Kumar A, McGovern EM, (2017): In vivo imaging of translocator protein, a marker of activated microglia, in alcohol dependence. Mol Psychiatry. 22:1759–1766. 28242869 10.1038/mp.2017.10 PMC5573660

[55] Hillmer AT, Angarita GA, Esterlis I, Anderson JM, Nabulsi N, Lim K, (2021): Longitudinal imaging of metabotropic glutamate 5 receptors during early and extended alcohol abstinence. Neuropsychopharmacology. 46:380–385. 32919411 10.1038/s41386-020-00856-9 PMC7852514

[56] Ichise M, Toyama H, Innis RB, Carson RE (2002): Strategies to improve neuroreceptor parameter estimation by linear regression analysis. J Cereb Blood Flow Metab. 22:1271–1281. 12368666 10.1097/01.WCB.0000038000.34930.4E

[57] Mayo LM, Asratian A, Lindé J, Morena M, Haataja R, Hammar V, (2020): Elevated Anandamide, Enhanced Recall of Fear Extinction, and Attenuated Stress Responses Following Inhibition of Fatty Acid Amide Hydrolase: A Randomized, Controlled Experimental Medicine Trial. Biological Psychiatry. 87:538–547. 31590924 10.1016/j.biopsych.2019.07.034

[58] Valverde O, Noble F, Beslot F, Dauge V, Fournie-Zaluski MC, Roques BP (2001): Delta9-tetrahydrocannabinol releases and facilitates the effects of endogenous enkephalins: reduction in morphine withdrawal syndrome without change in rewarding effect. Eur J Neurosci. 13:1816–1824. 11359533 10.1046/j.0953-816x.2001.01558.x

[59] Koch M, Varela L, Kim JG, Kim JD, Hernandez-Nuno F, Simonds SE, (2015): Hypothalamic POMC neurons promote cannabinoid-induced feeding. Nature. 519:45–50. 25707796 10.1038/nature14260 PMC4496586

[60] Molaei M, Fatahi Z, Zaringhalam J, Haghparast A (2016): CB1 Cannabinoid Agonist (WIN55,212–2) Within the Basolateral Amygdala Induced Sensitization to Morphine and Increased the Level of mu-Opioid Receptor and c-fos in the Nucleus Accumbens. J Mol Neurosci. 58:446–455. 26803309 10.1007/s12031-016-0716-9

[61] Fattore L, Vigano D, Fadda P, Rubino T, Fratta W, Parolaro D (2007): Bidirectional regulation of mu-opioid and CB1-cannabinoid receptor in rats self-administering heroin or WIN 55,212–2. Eur J Neurosci. 25:2191–2200. 17419755 10.1111/j.1460-9568.2007.05470.x

[62] Caille S, Alvarez-Jaimes L, Polis I, Stouffer DG, Parsons LH (2007): Specific alterations of extracellular endocannabinoid levels in the nucleus accumbens by ethanol, heroin, and cocaine self-administration. J Neurosci. 27:3695–3702. 17409233 10.1523/JNEUROSCI.4403-06.2007 PMC6672416

[63] Sustkova-Fiserova M, Charalambous C, Havlickova T, Lapka M, Jerabek P, Puskina N, (2017): Alterations in Rat Accumbens Endocannabinoid and GABA Content during Fentanyl Treatment: The Role of Ghrelin. Int J Mol Sci. 18. 10.3390/ijms18112486 PMC5713452 29165386

[64] Zhang J, Wang N, Chen B, Wang Y, He J, Cai X, (2016): Blockade of Cannabinoid CB1 receptor attenuates the acquisition of morphine-induced conditioned place preference along with a downregulation of ERK, CREB phosphorylation, and BDNF expression in the nucleus accumbens and hippocampus. Neurosci Lett. 630:70–76. 27461790 10.1016/j.neulet.2016.07.047

[65] Jin L, Pan L, Guo Y, Zheng Y, Nie Z, Zhu R (2014): Expression and localization of cannabinoid receptor 1 in rats’ brain treated with acute and repeated morphine. Acta Neurobiol Exp (Wars). 74:288–297. 25231848 10.55782/ane-2014-1994

[66] Wiese B, Wilson-Poe AR (2018): Emerging Evidence for Cannabis’ Role in Opioid Use Disorder. Cannabis Cannabinoid Res. 3:179–189. 30221197 10.1089/can.2018.0022 PMC6135562

[67] Chye Y, Christensen E, Solowij N, Yucel M (2019): The Endocannabinoid System and Cannabidiol’s Promise for the Treatment of Substance Use Disorder. Front Psychiatry. 10:63. 30837904 10.3389/fpsyt.2019.00063 PMC6390812

[68] Sloan ME, Gowin JL, Ramchandani VA, Hurd YL, Le Foll B (2017): The endocannabinoid system as a target for addiction treatment: Trials and tribulations. Neuropharmacology. 124:73–83. 28564576 10.1016/j.neuropharm.2017.05.031

[69] Solinas M, Panlilio LV, Tanda G, Makriyannis A, Matthews SA, Goldberg SR (2005): Cannabinoid agonists but not inhibitors of endogenous cannabinoid transport or metabolism enhance the reinforcing efficacy of heroin in rats. Neuropsychopharmacology. 30:2046–2057. 15870833 10.1038/sj.npp.1300754

[70] Lichtman AH, Sheikh SM, Loh HH, Martin BR (2001): Opioid and cannabinoid modulation of precipitated withdrawal in delta(9)-tetrahydrocannabinol and morphine-dependent mice. J Pharmacol Exp Ther. 298:1007–1014. 11504797

[71] Yamaguchi T, Hagiwara Y, Tanaka H, Sugiura T, Waku K, Shoyama Y, (2001): Endogenous cannabinoid, 2-arachidonoylglycerol, attenuates naloxone-precipitated withdrawal signs in morphine-dependent mice. Brain Res. 909:121–126. 11478928 10.1016/s0006-8993(01)02655-5

[72] Lofwall MR, Babalonis S, Nuzzo PA, Elayi SC, Walsh SL (2016): Opioid withdrawal suppression efficacy of oral dronabinol in opioid dependent humans. Drug Alcohol Depend. 164:143–150. 27234658 10.1016/j.drugalcdep.2016.05.002 PMC4910823

[73] Hurd YL, Spriggs S, Alishayev J, Winkel G, Gurgov K, Kudrich C, (2019): Cannabidiol for the Reduction of Cue-Induced Craving and Anxiety in Drug-Abstinent Individuals With Heroin Use Disorder: A Double-Blind Randomized Placebo-Controlled Trial. Am J Psychiatry. 176:911–922. 31109198 10.1176/appi.ajp.2019.18101191

[74] Degenhardt L, Lintzeris N, Campbell G, Bruno R, Cohen M, Farrell M, (2015): Experience of adjunctive cannabis use for chronic non-cancer pain: findings from the Pain and Opioids IN Treatment (POINT) study. Drug Alcohol Depend. 147:144–150. 25533893 10.1016/j.drugalcdep.2014.11.031

[75] Campbell G, Hall WD, Peacock A, Lintzeris N, Bruno R, Larance B, (2018): Effect of cannabis use in people with chronic non-cancer pain prescribed opioids: findings from a 4-year prospective cohort study. Lancet Public Health. 3:e341–e350. 29976328 10.1016/S2468-2667(18)30110-5 PMC6684473

[76] Boehnke KF, Litinas E, Clauw DJ (2016): Medical Cannabis Use Is Associated With Decreased Opiate Medication Use in a Retrospective Cross-Sectional Survey of Patients With Chronic Pain. J Pain. 17:739–744. 27001005 10.1016/j.jpain.2016.03.002

[77] Haroutounian S, Ratz Y, Ginosar Y, Furmanov K, Saifi F, Meidan R, (2016): The Effect of Medicinal Cannabis on Pain and Quality-of-Life Outcomes in Chronic Pain: A Prospective Open-label Study. Clin J Pain. 32:1036–1043. 26889611 10.1097/AJP.0000000000000364

[78] Kral AH, Wenger L, Novak SP, Chu D, Corsi KF, Coffa D, (2015): Is cannabis use associated with less opioid use among people who inject drugs? Drug Alcohol Depend. 153:236–241. 26051162 10.1016/j.drugalcdep.2015.05.014 PMC4509857

[79] Bellnier T, Brown GW, Ortega TR (2018): Preliminary evaluation of the efficacy, safety, and costs associated with the treatment of chronic pain with medical cannabis. Ment Health Clin. 8:110–115. 29955555 10.9740/mhc.2018.05.110 PMC6007634

[80] Goggin MM, Shahriar BJ, Stead A, Janis GC (2019): Reduced urinary opioid levels from pain management patients associated with marijuana use. Pain Manag. 9:441–447. 31496363 10.2217/pmt-2019-0017

[81] Vigil JM, Stith SS, Adams IM, Reeve AP (2017): Associations between medical cannabis and prescription opioid use in chronic pain patients: A preliminary cohort study. PLoS One. 12:e0187795. 29145417 10.1371/journal.pone.0187795 PMC5690609

[82] El-Mourad J, Lunghi C, Herrera NP, Zongo A (2024): Dosing of cannabinoids associated with an opioid-sparing effect: a systematic review of longitudinal studies. Pain Management Nursing. 25:e8–e20. 37689509 10.1016/j.pmn.2023.08.005

[83] Nielsen S, Picco L, Murnion B, Winters B, Matheson J, Graham M, (2022): Opioid-sparing effect of cannabinoids for analgesia: an updated systematic review and meta-analysis of preclinical and clinical studies. Neuropsychopharmacology. 47:1315–1330. 35459926 10.1038/s41386-022-01322-4 PMC9117273

[84] Costa GPA, Nunes JC, Heringer DL, Anand A, De Aquino JP (2024): The impact of cannabis on non-medical opioid use among individuals receiving pharmacotherapies for opioid use disorder: a systematic review and meta-analysis of longitudinal studies. Am J Drug Alcohol Abuse. 50:12–26. 38225727 10.1080/00952990.2023.2287406

[85] Mayo LM, Asratian A, Lindé J, Morena M, Haataja R, Hammar V, (2020): Elevated Anandamide, Enhanced Recall of Fear Extinction, and Attenuated Stress Responses Following Inhibition of Fatty Acid Amide Hydrolase: A Randomized, Controlled Experimental Medicine Trial. Biol Psychiatry. 87:538–547. 31590924 10.1016/j.biopsych.2019.07.034

[86] D’Souza DC, Cortes-Briones J, Creatura G, Bluez G, Thurnauer H, Deaso E, (2019): Efficacy and safety of a fatty acid amide hydrolase inhibitor (PF-04457845) in the treatment of cannabis withdrawal and dependence in men: a double-blind, placebo-controlled, parallel group, phase 2a single-site randomised controlled trial. Lancet Psychiatry. 6:35–45. 30528676 10.1016/S2215-0366(18)30427-9

[87] Melis M, Pillolla G, Luchicchi A, Muntoni AL, Yasar S, Goldberg SR, (2008): Endogenous fatty acid ethanolamides suppress nicotine-induced activation of mesolimbic dopamine neurons through nuclear receptors. J Neurosci. 28:13985–13994. 19091987 10.1523/JNEUROSCI.3221-08.2008 PMC3169176

[88] Panlilio LV, Justinova Z, Goldberg SR (2013): Inhibition of FAAH and activation of PPAR: new approaches to the treatment of cognitive dysfunction and drug addiction. Pharmacol Ther. 138:84–102. 23333350 10.1016/j.pharmthera.2013.01.003 PMC3662489

[89] Miliano C, Natividad LA, Quello S, Stoolmiller M, Gregus AM, Buczynski MW, (2024): The Predictive Value of Plasma Bioactive Lipids on Craving in Human Volunteers With Alcohol Use Disorder. Biol Psychiatry Glob Open Sci. 4:100368. 39282655 10.1016/j.bpsgos.2024.100368 PMC11400622

[90] Boachie N, Gaudette E, Bazinet RP, Lin L, Tyndale RF, Mansouri E, (2023): Circulating Endocannabinoids and N-Acylethanolamines in Individuals with Cannabis Use Disorder-Preliminary Findings. Brain Sci. 13. 10.3390/brainsci13101375 PMC10605789 37891745

[91] Voegel CD, Kroll SL, Schmid MW, Kexel AK, Baumgartner MR, Kraemer T, (2022): Alterations of Stress-Related Glucocorticoids and Endocannabinoids in Hair of Chronic Cocaine Users. Int J Neuropsychopharmacol. 25:226–237. 34676867 10.1093/ijnp/pyab070 PMC8929753

[92] Mitra S, Gobira PH, Werner CT, Martin JA, Iida M, Thomas SA, (2021): A role for the endocannabinoid enzymes monoacylglycerol and diacylglycerol lipases in cue-induced cocaine craving following prolonged abstinence. Addict Biol. 26:e13007. 33496035 10.1111/adb.13007 PMC11000690

[93] Parsons LH, Hurd YL (2015): Endocannabinoid signalling in reward and addiction. Nat Rev Neurosci. 16:579–594. 26373473 10.1038/nrn4004 PMC4652927

[94] Solinas M, Goldberg SR, Piomelli D (2008): The endocannabinoid system in brain reward processes. Br J Pharmacol. 154:369–383. 18414385 10.1038/bjp.2008.130 PMC2442437

[95] Hirvonen J, Zanotti-Fregonara P, Umhau JC, George DT, Rallis-Frutos D, Lyoo CH, (2013): Reduced cannabinoid CB1 receptor binding in alcohol dependence measured with positron emission tomography. Mol Psychiatry. 18:916–921. 22776901 10.1038/mp.2012.100 PMC3594469

[96] Hirvonen J, Zanotti-Fregonara P, Gorelick DA, Lyoo CH, Rallis-Frutos D, Morse C, (2018): Decreased Cannabinoid CB(1) Receptors in Male Tobacco Smokers Examined With Positron Emission Tomography. Biol Psychiatry. 84:715–721. 30121138 10.1016/j.biopsych.2018.07.009 PMC6388688

[97] Farquhar CE, Breivogel CS, Gamage TF, Gay EA, Thomas BF, Craft RM, (2019): Sex, THC, and hormones: Effects on density and sensitivity of CB1 cannabinoid receptors in rats. Drug and alcohol dependence. 194:20–27. 30391834 10.1016/j.drugalcdep.2018.09.018 PMC6312486

[98] Liu X, Li X, Zhao G, Wang F, Wang L (2020): Sexual dimorphic distribution of cannabinoid 1 receptor mRNA in adult C57BL/6J mice. Journal of Comparative Neurology. 528:1986–1999. 31997354 10.1002/cne.24868

[99] Llorente-Berzal A, Assis MA, Rubino T, Zamberletti E, Marco EM, Parolaro D, (2013): Sex-dependent changes in brain CB1R expression and functionality and immune CB2R expression as a consequence of maternal deprivation and adolescent cocaine exposure. Pharmacological research. 74:23–33. 23680694 10.1016/j.phrs.2013.05.001

[100] Paola Castelli M, Fadda P, Casu A, Sabrina Spano M, Casti A, Fratta W, (2014): Male and female rats differ in brain cannabinoid CB1 receptor density and function and in behavioural traits predisposing to drug addiction: effect of ovarian hormones. Current pharmaceutical design. 20:2100–2113. 23829370 10.2174/13816128113199990430

[101] Craft RM, Wakley AA, Tsutsui KT, Laggart JD (2012): Sex differences in cannabinoid 1 vs. cannabinoid 2 receptor-selective antagonism of antinociception produced by Δ9-tetrahydrocannabinol and CP55, 940 in the rat. Journal of Pharmacology and Experimental Therapeutics. 340:787–800. 22182934 10.1124/jpet.111.188540

[102] Van Laere K, Goffin K, Casteels C, Dupont P, Mortelmans L, de Hoon J, (2008): Gender-dependent increases with healthy aging of the human cerebral cannabinoid-type 1 receptor binding using [18F] MK-9470 PET. Neuroimage. 39:1533–1541. 18077184 10.1016/j.neuroimage.2007.10.053

[103] Neumeister A, Normandin MD, Pietrzak RH, Piomelli D, Zheng M-Q, Gujarro-Anton A, (2013): Elevated brain cannabinoid CB1 receptor availability in post-traumatic stress disorder: a positron emission tomography study. Molecular psychiatry. 18:1034–1040. 23670490 10.1038/mp.2013.61 PMC3752332

[104] Normandin MD, Zheng M-Q, Lin K-S, Mason NS, Lin S-F, Ropchan J, (2015): Imaging the cannabinoid cb1 receptor in humans with [11c] omar: assessment of kinetic analysis methods, test–retest reproducibility, and gender differences. Journal of Cerebral Blood Flow & Metabolism. 35:1313–1322. 25833345 10.1038/jcbfm.2015.46 PMC4528005

[105] Laurikainen H, Tuominen L, Tikka M, Merisaari H, Armio R-L, Sormunen E, (2019): Sex difference in brain CB1 receptor availability in man. Neuroimage. 184:834–842. 30296558 10.1016/j.neuroimage.2018.10.013

[106] Radhakrishnan R, Worhunsky PD, Zheng M-Q, Najafzadeh S, Gallezot J-D, Planeta B, (2022): Age, gender and body-mass-index relationships with in vivo CB1 receptor availability in healthy humans measured with [11C] OMAR PET. NeuroImage. 264:119674. 36243269 10.1016/j.neuroimage.2022.119674

